# Dynamic *in vivo *imaging and cell tracking using a histone fluorescent protein fusion in mice

**DOI:** 10.1186/1472-6750-4-33

**Published:** 2004-12-24

**Authors:** Anna-Katerina Hadjantonakis, Virginia E Papaioannou

**Affiliations:** 1 Department of Genetics and Development, College of Physicians and Surgeons of Columbia University, 701 West 168^th ^St., New York, NY 10032, USA; 2 Developmental Biology Program, Sloan-Kettering Institute, New York, NY10021, USA

## Abstract

**Background:**

Advances in optical imaging modalities and the continued evolution of genetically-encoded fluorescent proteins are coming together to facilitate the study of cell behavior at high resolution in living organisms. As a result, imaging using autofluorescent protein reporters is gaining popularity in mouse transgenic and targeted mutagenesis applications.

**Results:**

We have used embryonic stem cell-mediated transgenesis to label cells at sub-cellular resolution *in vivo*, and to evaluate fusion of a human histone protein to green fluorescent protein for ubiquitous fluorescent labeling of nucleosomes in mice. To this end we have generated embryonic stem cells and a corresponding strain of mice that is viable and fertile and exhibits widespread chromatin-localized reporter expression. High levels of transgene expression are maintained in a constitutive manner. Viability and fertility of homozygous transgenic animals demonstrates that this reporter is developmentally neutral and does not interfere with mitosis or meiosis.

**Conclusions:**

Using various optical imaging modalities including wide-field, spinning disc confocal, and laser scanning confocal and multiphoton excitation microscopy, we can identify cells in various stages of the cell cycle. We can identify cells in interphase, cells undergoing mitosis or cell death. We demonstrate that this histone fusion reporter allows the direct visualization of active chromatin *in situ*. Since this reporter segments three-dimensional space, it permits the visualization of individual cells within a population, and so facilitates tracking cell position over time. It is therefore attractive for use in multidimensional studies of *in vivo *cell behavior and cell fate.

## Background

Macro- and microscopic imaging are pivotal readouts in the field of biology both for determining the normal (baseline) course of events and for observing the effects of experimental perturbations and natural aberrations [1]. Recent advances in microscopic imaging make it possible to routinely gain visual access to samples hundreds of microns thick [2]. The emergence of green fluorescent protein (GFP) as a reporter has opened up many new experimental approaches that were not previously possible [2-4]. GFP and other genetically-encoded autofluorescent protein reporters have a number of properties that make them ideal for multidimentional imaging of living specimens: no substrate (except photons) is required to generate signal, they have a high signal-to-noise ratio, are non-toxic, stable at 37°C and resistant to photobleaching. Moreover they are available in an increasingly large compendium of spectrally-distinct variants.

To construct high-resolution anatomical models of normal, mutant and pathological situations, we must establish technologies to identify and follow individual cells in three-dimensional (3D) space and in 3D over time, in four-dimensions (4D). Unfortunately, native fluorescent proteins permit tracking the position of any given cell over time only if the population of tagged cells is distributed among non-expressing cells by virtue of lineage or in a mosaic experimental situation [5-9]. In situations where groups, or all cells in a 3D field of view express a fluorescent protein label, information on the behavior of individual cells cannot be discerned. Therefore an approach is required where 3D space is segmented at cellular resolution. This is most easily achieved if each cell can be marked with an easily identifiable tag that is visible at subcellular resolution [10,11]. Since it exhibits low autofluorescence, and is a single, universal and volumetrically constrained cellular organelle, the nucleus is ideal for such labeling [12-14].

Our goal was to take advantage of this feature and to develop a non-invasive fluorescent protein marker of the nucleus for *in toto *imaging (all cells within the multidimensional space being imaged – discussed in Ref. [11]) of individual cells *in situ *in living mice [10-12]. For the unequivocal identification of individual cells, we sought a developmentally neutral, genetically-encoded autofluorescent protein-based marker that labels DNA during all phases of the cell cycle while preserving cell morphology and behavior.

As the principal structural proteins of eukaryotic chromosomes, histones are attractive targets for fluorescent nuclear labeling. Histone tagged fluorescent protein fusions have previously been shown to incorporate into chromatin without any adverse effects on the viability of cells in culture [15]. When compared to reporters containing nuclear localization sequences (nls), histone fusions exhibit an improved signal-to-noise ratio and have the distinct advantage of signal remaining bound to the target even during cell division when the nuclear envelope has broken down. In contrast nls-tagged markers (both GFP and lacZ) become dispersed throughout a cell during division, making it difficult to distinguish individual cells during mitosis.

To date GFP fusions to several histones have been generated and used for labeling nuclei in live transgenic animals, including nematode worms, fruit flies and zebrafish [13,14,16,17]. One of these is a fusion between EGFP and human histone H2B which was developed in order to label active chromatin and used to follow the segregation of double minute chromosomes in cancer cells [15]. We have investigated the expression and germline transmission of this type of fusion in mice and established its usefulness not only for imaging cell cycle dynamics [18], but also for tracking cells in living specimens. Moreover unlike native GFP variants this subcellularly localized histone fusion was found to withstand fixation while retaining both fluorescence and subcellular localization.

## Results

To evaluate histone-tagged fluorescent protein fusions in embryonic stem (ES) cells and mice, we generated constructs comprising an N-terminally positioned human H2B sequence followed at the C-terminus by sequences for various fluorescent proteins both GFP and DsRed-based. We previously reported that DsRed1 was not amenable to use in ES cells or mice [22], however several improved DsRed variants have recently become available [10]. We therefore chose to evaluate DsRed2 and DsRedExpress as part of this study.

The H2B-fluorescent protein fusions we generated were introduced into vectors utilizing the CAG promoter [19] designed to drive high-level constitutive gene expression in ES cells, embryos and adult mice [20]. Standard protocols were used to establish stable lines of ES cells constitutively expressing an H2B fusion [20-22]. Several transgenic ES cell lines were generated each expressing H2B-EGFP at strong homogenous levels [23]. However, even though we did recover lines with H2B-DsRed2 and H2B-DsRedExpress expression [24], subsequent maintenance of these lines in culture revealed a continued reduction and heterogeneity in fluorescence. We were unable to establish lines with sustained homogenous H2B-DsRed2 or H2B-DsRedExpress fluorescence. Moreover our recent data suggest that mRFP1 [10,25], a rapidly-maturing monomeric form of DsRed, is amenable to use in mice, both in its native form and as a part of functional fusion proteins (AKH unpublished observations).

**Figure 1:**
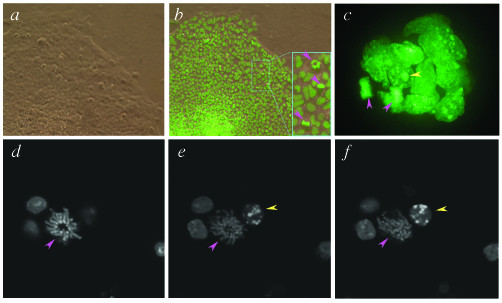
**Imaging chromatin in living transgenic ES cells constitutively expressing a H2B-EGFP fusion protein. **(*a*) Bright-field and (*b*) dark-field micrographs of a CAG::H2B-EGFP ES cell colony. The inset shows a detail with three nuclei in metaphase (pink arrowheads) with the metaphase plates orientated differently. The mitotic spindle of the cell at the top is closely aligned to the *z*-*y *plane whereas those for the lower two cells are more closely aligned with the *x*-*z *planes. (*c*) Rendered stack (3-D reconstruction) of sequential optical slices acquired using spinning disc confocal methodology, projected as a fixed angle view of an embryoid body comprised of ES cells constitutively expressing a H2B-EGFP fusion. Pink arrowheads indicate two nuclei in late-anaphase – telophase. Yellow arrowhead points to the nuclear remnant of a cell that has necrosed or apoptosed. (*d *– *f*) High-power sequential optical sections each (1 μm apart) through ES cells constitutively expressing the H2B-EGFP fusion, taken using laser scanning confocal methodology showing interphase nuclei, a mitotic nucleus (pink arrowhead) and a pycnotic nucleus (yellow arrowhead).

**Figure 2:**
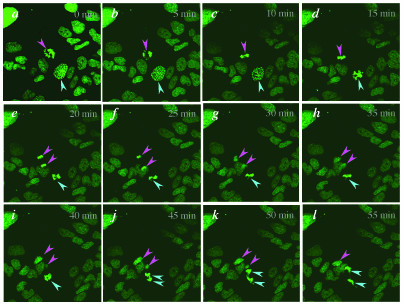
**Live imaging the progression through mitosis. **Laser scanning confocal *x*-*y *images taken at a single *z*-plane at five minute intervals for one hour. Note that not all green fluorescence (corresponding to nuclear material) will be represented in the plane being imaged. A cell progressing from anaphase to cytokinesis (pink arrowheads). A cell progressing from prophase to telophase (blue arrowheads). The average time taken to transition from early prophase to cytokinesis was calculated to be approximately 1 hour (*n *= 30).

H2B-EGFP expressing ES cells are shown in Fig. 1. It noteworthy that with this histone fusion we observed a high signal-to-noise ratio and so could achieve high-resolution imaging of mitotic chromosomes (pink arrowheads), various states of interphase chromatin and nuclear debris (yellow arrowheads). Moreover for cells undergoing mitosis we could also discern the stage of mitosis and the plane of cell division (Fig. 1b inset). Previous work indicated that a similar fusion protein expressed in HeLa cells did not affect cell cycle progression [15], and accordingly not only could we visualize nuclear dynamics and identify the various phases of mitosis in live ES cells [26] (Fig. 2), but in doing so, we did not observe any change in growth rate or mitotic index in the transgenic ES cells compared to non-transgenic parental ES cells (data not shown). By imaging several CAG::H2B-EGFP transgenic ES cells undergoing mitosis (*n *= 30) we calculated the progression from early prophase to cytokinesis to take less than one hour (Fig. 2). Furthermore imaging of embryoid bodies demonstrated that individual nuclei could be discerned from a three-dimensional population of densely packed cells all of which were expressing the H2B-EGFP marker (Fig. 1c). No loss of fluorescence was observed with prolonged *in vitro *passage of the ES cells expressing the H2B-EGFP fusion in the absence of positive selection in the presence or absence of LIF (*t *> 3 months in the presence of LIF).

We next tested the effects of widespread expression of an H2B fusion protein in mice. We generated germ line chimeras and established transgenic lines of mice constitutively expressing H2B-EGFP. We were able to breed this transgene to homozygosity, resulting in viable and fertile animals exhibiting widespread expression with no overt morphological abnormalities. The transgene has been maintained for over three years in a breeding colony of homozygous mice with no apparent effect on viability, breeding performance or lifespan. We therefore infer that this fusion protein is developmentally neutral and does not interfere with either mitosis or meiosis. Wide-field microscopic analysis of both mouse embryos and adult organs demonstrates widespread expression of the H2B-EGFP fusion in all types of nucleated cells.

**Figure 3:**
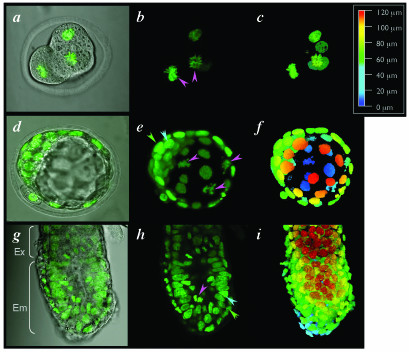
**Live embryo imaging of preimplantation and early postimplantation mouse embryos hemizygous for a constitutively expressed H2B-EGFP fluorescent fusion. ***(a) *Single confocal optical section fluorescence overlay on a bright-field image of a 5-cell stage pre-implantation embryo. Two of the blastomeres are dividing synchronously and are in metaphase (pink arrowheads in b). *(b) *Dark-field projection of the entire rendered *z*-stack of *x*-*y *sections (n = 19), through the entire embryo shown in panel *a*. *(c) *Color-coded depth projection of the entire *z*-stack of *x*-y images for the embryo shown in the previous panels. (*d*) Single confocal optical section fluorescence overlay on a bright-field image of a blastocyst stage embryo. Inner cell mass (ICM) is to the top left corner and second polar body is on the bottom left, juxtaposed to the edge of the ICM. (*e*) Dark-field projection of half the rendered *z*-stack of *x*-*y *sections (*n *= 40, sections 1–19 were used for generating the projection), spanning half the embryo shown in panel *d*. Condensed chromosomes of nuclei in prophase (pink arrowheads) can be seen in three cells of the mural trophectoderm. Cells of the polar trophectoderm (green arrowhead) and inner cell mass (blue arrowhead) can also be distinguished by position within the half-blastocyst reconstruction. (*f*) Color-coded depth projection of the entire *z*-stack of *x*-y images for the embryo shown in the previous two panels. (*g*-*h*) Saggital views and rendered *z*-stacks of *x*-*y *images of an E5.75 (pre-streak stage) embryo. (*g*) Single optical confocal section fluorescence overlay on a bright-field image positioned half the way through the embryo. The brackets on the left illustrate the position of the embryonic (Em) and extraembryonic (Ex) regions of the embryo. (*h*) The same optical section with only the fluorescence image. Cells of the epiblast (blue arrowhead) and visceral endoderm (green arrowhead) can clearly be distinguished on the basis of position and nuclear morphology. Cells in mitosis can readily be distinguished within the embryo (pink arrowhead). (*i*) Color-coded depth projection of the stack of serial sections (*n *= 60), part of the series of which is shown in the previous two panels. Color-coded *z*-scale (upper right) applies to all projections and denotes distances along the *z*-axis (0–120 μm).

We used laser scanning confocal microscopy [10,11] to image this constitutively expressed transgenic reporter at subcellular resolution in live mouse embryos. Such non-invasive visualization of chromatin in living preparations allowed us to acquire high-magnification sequential optical sections (*z*-stacks) that can be used to generate high-resolution anatomical volumetric (3-dimensional) images with details of interphase chromatin in addition to mitotic chromosomes and fragmenting nuclei. To do this, stacks of sequential optical sections are reconstructed into 3-dimensional projections. This methodology can be used to generate 3-dimensional (3D) image sets not only of cells propagated in culture but also of cells *in situ *in living animals and is illustrated here by imaging whole mouse embryos at the 4-cell stage, the blastocyst stage, and the pre-gastrula stage (Fig. 3 and Additional Files 1 and 2). These data sets can be computationally manipulated in various ways, for example for the visualization of individual *xy *slices from a *z*-stack, rendered images from the full, or part of a *z*-stack, and color-coded depth projections of a *z*-stack (Fig. 3).

Data on older embryos and adult organs illustrates that larger specimens can be imaged, however not in their entirety given current limitations in optical imaging capabilities. Instead of imaging the whole specimen, larger samples are positioned so that data can be acquired from regions of interest, which can then be acquired in a tiled manner and computationally re-aligned in image acquisition and processing software.

**Figure 4:**
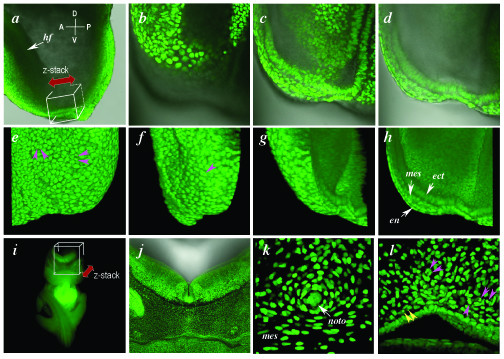
**Live imaging H2B-EGFP in postimplantation mouse embryos. ***(a) *Lateral view of the embryonic region of an E7.5 embryo (anterior to the left) with box depicting the region imaged in *b *and double-headed arrow depicting the *x*-*y *layering of the *z*-stack. (*b*-*d*) single optical *x*-*y *sections of fluorescence overlayed on bright-field images acquired at the same focal plane. Each panel is 60 μm apart from the preceding panel. These panels comprise *x*-*y *images in the *z*-stack depicted in panel *a*. The different layers of this stage of embryo including the epiblast, mesoderm, visceral endoderm and node can be distinguished on the basis of both position and nuclear morphology. (*e*-*h*) projection of a rendered *z*-stack of (*x*-*y*) sections (*n *= 90) of the dark-field component of the sections taken in the series schematized in *a *and of the raw data shown in *b*. (*e*) 0° rotation, (*f*) 60° rotation, (*g*) 120° rotation, and (*h*) 180° rotation views. (*i*) low-magnification frontal view of an E11 embryo that has had a transverse cut made to remove the head. The box depicts the region (at the ventral hindbrain and 1^st ^branchial pouch) subject to laser scanning confocal imaging, with the double-headed arrow depicting the *x*-*y *layering of the acquired *z*-stack. (*j*) rendered (*z*-) stack of sections (*n *= 200, *i.e*. 400 μm depth) taken through the boxed region. (*k*) rendered stack of top 50 *x*-*y *sections (100 μm depth) taken from the region imaged around the notochord (comprising axial mesoderm and mesenchyme cells). (*l*) rendered stack of top 50 sections (100 μm depth) taken around the branchial pouch region (comprising endoderm and mesenchyme cells). The sections used to generate the rendered stacks in panels *k *and *l *were electronically magnified. Pink arrowheads, mitotic nuclei; yellow arrowheads, pycnotic nuclei; ect, ectoderm, en, endoderm, hf, headfold, mes, mesoderm, noto, notochord.

Our data demonstrates that nuclear morphology afforded by the H2B-EGFP fusion can be used to identify different cell types. In both the raw data, and a rotated rendered stack of an embryonic day (E) 7.5 embryo, cells of the definitive endoderm, mesoderm and embryonic ectoderm can be distinguished solely on the basis of nuclear morphology and orientation in addition to their expected position (Fig. 4b–h and Additional File 3). Low magnification rendered *z*-stacks taken from a transversely cut section through the head of an E10.5 embryo (Fig. 4i) reveal the stereotypical 3D organization of nuclei within the region imaged (Fig. 4j), and electronically magnified views of this image illustrate a characteristic apposition of nuclei both in and around the notochord, and within the mesenchyme and endoderm of the pharyngeal region (Fig. 4k and 4l and Additional Files 4 and 5), in addition to providing information on cell division and cell death (pink and yellow arrowheads, respectively in Fig. 4l).

**Figure 5:**
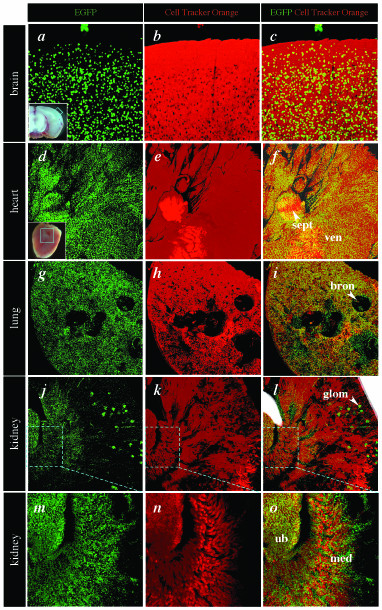
**High resolution live imaging of the organs of CAG::H2B-EGFP adult mice. **Confocal images of freshly isolated organs from a 6 week old adult male hemizygous CAG::H2B-EGFP Tg/+ animal illustrate the widespread nuclear localized expression of the histone fusion. A transverse cut was made through each organ and the cut surface was placed closest to the objective lens and imaged. Cell tracker orange was used as a vital cytoplasmic counter stain. The panels show rendered confocal *z*-stacks imaged through 80 μm of the brain using a 20x plan-apo objective (*a*-*c*), 568 μm of the heart using a 5x fluar objective (*d*-*f*), 142 μm of a lung lobe using a 5x fluar objective (*g*-*i*) and 346 μm of a kidney using a 5x fluar objective low power view (*j*-*l*), and high power view (*m*-*o*). Insets in panels *a *and *d *show the region of the brain and heart imaged, respectively. High resolution images of the kidney (*m*-*o*) illustrate electronic magnification of the data shown in *j*-*l*. Bron, bronchus; glom, glomeruus; med, medulla; sept, septum; ub, ureteric bud; ven, ventricle. Areas of increased fluorescence in the red channel are an artefact due to saturated pixels in regions of the sample closest to the objective.

Wide-field microscopic examination of organs from adult animals revealed widespread fluorescence as has been reported for animals expressing native fluorescent proteins under the regulation of the CAG promoter [20,22,27]. Laser scanning confocal imaging of various organs obtained from adult animals was used to generate high-resolution images revealing stereotypical nuclear positions, reflecting different cell types and revealing other subcellular details, such as mitosis and nuclear fragments, also observed in embryos (Fig. 5 and Additional Files 3, 4, 5).

**Figure 6:**
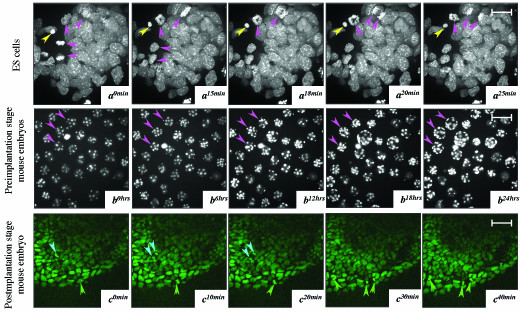
**Dynamic time-lapse imaging of mouse CAG::H2B-EGFP transgenic ES cells, preimplantation and postimplantation embryos using different imaging modalities. **(*a*) Rendered confocal stacks of transgenic ES cells constitutively expressing a CAG::H2B-EGFP transgene representing a 25 minute time-lapse recording of images acquired using a spinning disc confocal scan head. *x*-*y *sections with a *z*-interval of 0.2 μm were taken at a rate of 10/second over a total *z*-stack of 40 μm. Cells can be traced through the 4D rendered stack. Cells entering or completing mitosis (pink arrowheads) and the nuclear remnant of a cell that has either undergone apoptosis or necrosis (yellow arrowhead) are clearly visible. (*b*) Wide-field imaging of CAG::H2B-EGFP transgenic preimplantation embryos. This 24 hour image sequence illustrates cavitation leading up to the formation of the blastocyst in several embryos (violet arrowheads). (*c*) Rendered two-photon stacks of CAG::H2B-EGFP transgenic gastrulation stage postimplantation embryos. This 40 minute time-lapse sequence illustrates cell division and tracking within the visceral endoderm (green arrowhead) and epiblast (blue arrowheads) and the movement of mesoderm emanating from the primitive streak, which is positioned to the right, out of the field of view. Scale bar in *a *= 10 μm, *b *= 100 μm and *c *= 50 μm.

Finally we investigated whether we could follow cell movement, division, and death in time-lapse experiments using various imaging modalities. We cultured ES cells and embryos on the stages various each of which had been modified to permit culture under physiological conditions. The different types of data routinely generated using different optical imaging modalities that are widely used and commercially available are illustrated in Figure 6. Spinning disc confocal microscopy [1] was used for short-term high-resolution 4D imaging of CAG::H2B-EGFP ES cells (Fig. 6 and Additional File 6), wide-field microscopy was used for long-term low-resolution imaging of CAG::H2B-EGFP preimplantation stage embryos (Fig. 6 and Additional File 7). Note that development proceeds normally in most embryos, and that some of the embryos imaged are undergoing cavitation to form blastocysts [28] (arrowheads). Two-photon excitation microscopy [10,29,30] was used to image cells in a whole gastrula-stage mouse embryo without perturbing the morphogenetic movements associated with gastrulation (Fig. 6 and Additional File 8). Cells can clearly be followed through the successive time points in each of these experimental situations ranging from a few minutes (short-term) to 24 hours (long-term) time-lapse duration. These studies reflect the range of resolutions at which information can be acquired using a marker of this type. We observed normal cell proliferation throughout the course of these imaging experiments and no excessive nuclear fragmentation. Also, because the on-stage cultures were comparable to parallel cultures maintained in a tissue culture incubator, we conclude that the outcome of the cultures was not affected by the various imaging modalities.

## Discussion

Here we report the evaluation of a chromatin localized histone fusion fluorescent reporter *in vivo *through the generation of transgenic embryonic stem (ES) cells and mice having widespread expression of this reporter.

The transgenic mice that we have generated provide a new tool for high-resolution live imaging of a genetically tractable mammalian model organism [10,11]. They represent a resource for analyzing development and disease at the subcellular level in cells, embryos and adult tissues. The marker used facilitates the acquisition of *in vivo *data and allows it to be integrated onto a high-resolution anatomical framework. This type of multidimensional data is complex and thus difficult to digitize and compile into a standardized and integratable format. *In toto *imaging of fluorescent protein expressing specimens on a large-scale could be used for generating high-resolution digitally recorded anatomical databases where the baseline (wild-type) cell behaviors and cell fates can be contrasted to those observed in mouse mutants. However, developing *in toto *imaging technologies for acquiring large amounts of data will necessitate improving the speed and throughput of microscopic image acquisition and analysis. This would also be coincident with the ongoing development of improved computational approaches to mine and integrate this type of data [discussed in ref. [11]].

**Figure 7:**
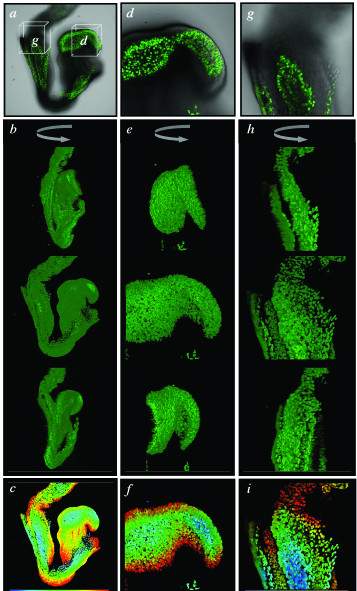
**High-resolution 3-dimensional imaging of fixed CAG::H2B-EGFP transgenic embryos. **Confocal images of an E8.5 CAG::H2B-EGFP transgenic embryo fixed in 4% paraformaldehyde for 72 hours, then washed, stored and imaged in PBS. Low-magnification views and reconstructions of whole embryo (*a*-*c*). Boxes in *a *designate region imaged in *d *and *g*. High-magnification views of the headfolds (*d*-*f*) and posterior primitive streak and proximal allantois (*g*-*i*). Single *xy *images (*a*, *d *and *g*) from the *z*-stacks used to computationally render the data sets. These images are overlayed onto the bright field channel so as to display the outline of the embryo. Rotations through the rendered *z*-stacks displayed at 45° intervals (*b*, *e *and *h*). Color-coded depth projections of each of the *z*-stacks (*c*, *f *and *i*).

Much of the information generated using a fluorescent fusion reporter such as the H2B-EGFP fusion is analogous to conventional histology [21,31] except that this mode of data acquisition optically sections a sample (circumventing the need to physical section), excels in permitting computational 3D reconstructions of spatial information, and can additionally be coupled to time-lapse imaging for the capture, processing and quantitation of 4D information (Fig. 6 and Additional Files). Also, unlike conventional GFP-based reporters [20,22], the histone H2B-EGFP fusion is resilient to fixation, so samples can be processed and stored for extended periods of time without compromising signal integrity or specificity (Fig. 7).

The future development and availability of mouse strains constitutively expressing spectral variant histone H2B fusions should prove useful for visualizing anatomy and tracking different populations of cells in multiple dimensions at high-resolution in mice as has previously been demonstrated in other organisms which are classically perceived as being more amenable to *in vitro *culture and optical imaging [13,16]. They can also be used as tagged populations of cells in chimeras [8], in addition to transplantation and cell isolation experiments [32]. Also, since fluorescence is proportional to genome content and the fluorescence intensity reflects chromosome condensation state, the reagents we have generated should permit the study of alterations in ploidy and chromosomal condensation including determination of phases of mitosis [26]. Additionally, real-time analysis of chromatin fragmentation as well as the effects of mutations on chromosome stability during disease processes can be investigated using CAG::H2B-EGFP transgenic mice.

## Conclusions

The CAG::H2B-EGFP strain that we have generated takes *in vivo *imaging using genetically-encoded reporters in mice to sub-cellular resolution. The development of additional strains permitting spectrally-distinct high-resolution live cell *in vivo *imaging, coupled to advances in optical imaging modalities and the development of improved computational methods to mine imaging data should pave the way for a multidimensional understanding of biological processes.

It is anticipated that in the future, *in vivo *imaging approaches using transgenic animals expressing genetically-encoded fluorescent proteins will not only provide high-resolution information on cell behavior in specific biological processes [12,33], but more importantly it may lead to an exponential increase in the available multidimensional *in vivo *biological information which could mirror the recent explosion of available genomic data. Therefore recent advances in live imaging will need to be paired with developments in computational biology, as appropriate informatics methods will need to be developed and implemented in order to mine, present and integrate this type of *in vivo *biological data.

## Methods

The coding sequence for the human histone H2B gene (X57127) was amplified from genomic DNA by PCR using *Pfx *Polymerase (Invitrogen). The resulting product was cloned into pCR4 TOPO (Invitrogen) to generate pH2B. The H2B fragment was then cloned into plasmids pEGFP-N1, pDsRed2-N1 pDsRedExpress-N1 (BD Biosciences, Inc) in order to generate plasmids pH2B-EGFP, pH2B-DsRed2 and pH2B-DsRedExpress (oligonucleotide sequences are available upon request). The resulting fusions were then re-amplified by PCR and cloned into the XhoI site of pCAGGS [19] to generate pCX-H2B-EGFP, pCX-H2B-DsRed2 and pCX-H2B-DsRedExpress. All vectors were tested by transient transfection of Cos-7 cells and R1 ES cells using Fugene 6 Transfection Reagent as per manufacturer's recommendations (Roche) and electroporation respectively. The H2B-DsRed2 and H2B-DsRedExpress fusions failed to produce sustained homogenous levels of fluorescent signal, however the H2B-EGFP fusion gave strong nuclear-localized fluorescence throughout the extended culture period without perturbing cell morphology, the rate of proliferation, or the mitotic index (Fig. 2 and data not shown).

Transgenic ES cell lines constitutively expressing H2B-EGFP were generated by co-electroporation of the linearized reporter construct and a circular PGK-Puro-pA plasmid [34] conferring transient puromycin resistance. Puromycin selection was carried out as described previously [20,22]. Briefly, drug selection was initiated 24–36 hours after electroporation, maintained for 5 days, after which time it was replaced with non-selection media for a further 24–48 hours. Fluorescent colonies were identified and picked under an epifluorescence microscope (Nikon SMZ1500). Clones were passaged in 96-well plates, and scored for maintenance and extent of fluorescence. Those exhibiting homogeneous and robust transgene expression *in vitro *under both stem cell conditions and conditions employed to promote their differentiation were maintained further. For stem cell conditions ES cells were grown on gelatin in the presence of LIF. For differentiation, ES cells were grown on bacteriological Petri dishes in the absence of LIF for 2–5 days to promote embryoid body formation. Thereafter embryoid bodies were re-plated onto tissue culture dishes in the presence of factors promoting directed differentiation.

To assess whether an H2B fusion can continue to be widely expressed and transmitted through the germline of mice we used H2B-EGFP expressing ES cells for chimera generation by injection into C57BL/6 blastocysts using standard procedures [21]. Chimeras were bred to outbred ICR and inbred 129/Tac mice (Taconic, Germantown, NY) for germline transmission and subsequent maintenance of the lines. Two independent clones were taken germline giving indistinguishable results. We therefore focused on one of the transgenic lines. After germline transmission, this transgene was maintained at homozygosity, suggesting that the site of integration is not perturbing essential gene function. All animals retained widespread homogenous fluorescence for at least five subsequent generations. Homozygotes were distinguished from heterozygotes either by increased fluorescence in newborn (unpigmented) animal tails, by breeding, or by intensity of an EGFP hybridizing fragment on a Southern blot.

Embryos and organs were dissected in HEPES buffered DMEM media containing 10% fetal calf serum, then cultured either in a standard tissue culture incubator or on a microscope stage under standard conditions promoting the culture of mouse embryos [35,36] in 50% rat serum: 50% DMEM buffered with bicarbonate and maintained under physiological conditions in a closed temperature-controlled, humidified and oxygenated (95% air, 5% CO_2_) chamber (Bioptechs Inc. or Solent Sci Ltd. or home-made). For cytoplasmic staining, samples were incubated in Cell Tracker Orange (Molecular Probes; 1:500 dilution in dissecting or culture media) for 10–20 minutes. Embryos were kept in a standard tissue culture incubator at 37°C during staining. Samples were then washed twice with warm dissecting or culture media prior to imaging.

All images shown (except Fig. 7) are of living hemizygous (Tg/+) embryos or freshly dissected (unfixed) tissues obtained from Tg/+ adults and maintained under physiological conditions. Increased fluorescence and a higher signal-to-noise ratio was observed in homozygous (Tg/Tg) specimens. The embryo presented in Figure 7 was fixed in 4% paraformaldehyde at 4°C for 72 hours, then washed, stored and imaged in PBS. Similar results were obtained in embryos fixed for up to two weeks.

Wide-field images were acquired on a Nikon SMZ1500 stereo-dissecting microscope or Nikon Eclipse 5000 inverted microscope equipped with epifluorescent illumination. Spinning disc confocal data was acquired on an UltraView RS3 (Perkin-Elmer Systems) fitted on a Zeiss Axiovert 200M microscope with illumination from a 488 nm Argon laser (Melles Griot). Laser scanning confocal and multiphoton excitation data were taken on a Zeiss LSM510 NLO on a Zeiss Axioscop 2 FS MOT microscope. Objective lenses used on the Axiovert 200M and Axioscop 2 were plan-apochromat 63x/NA1.4, C-apochromat 40x/NA1.2, a plan-apochromat 20x/NA0.75 and a fluar 5x/NA0.25. For laser scanning microscopy GFP was excited using either a 488 nm Argon laser (Lassos, Inc) at 488 nm (for single-photon excitation) or a Titanium:Sapphire laser (Coherent Mira 900F with Verdi 5W pump laser) tuned between 860 and 890 nm (for two-photon excitation). Cell Tracker Orange was excited using a 543 nm HeNe laser (for single-photon use).

Images were acquired as *z*-stacks comprising sequential *x*-*y *sections taken at 0.1–2 μm *z*-intervals. Raw data was processed using a variety of packages including Zeiss AIM software (Carl Zeiss Microsystems at ), Image J (NIH at ) and Volocity (Improvision at ). Each image series was re-animated using software to make the time-lapse movies that are available as additional files. Both rendered volume and time-lapse movies were assembled in QuickTime Player (Apple Computer, Inc at ).

## Appendix

The CAG::H2B-EGFP strain of mice generated in this study will be made available through the Jackson Laboratories Induced Mutant Resource (JAX IMR at ).

## Supplementary Material

Additional File 1 Rotating 3D projection of a whole live CAG::H2B-EGFP Tg/+ blastocyst. Supplementary to stills presented in Fig. 3. This file was assembled at 6 frames/second. Click here for file

Additional File 2 Rotating 3D projection of a (electronic) half blastocyst. Generated from the same raw data set used to compile Supplementary File 1. Supplementary to stills presented in Fig. 3. This file was assembled at 6 frames/second. Click here for file

Additional File 3 Rotating 3D projection the node region of a live CAG::H2B-EGFP Tg/+ E7.5 embryo. Supplementary to stills presented in Fig. 4. This file was assembled at 6 frames/second. Click here for file

Additional File 4 Rotating 3D projection of the notochord of a live CAG::H2B-EGFP Tg/+ E10.5 embryo. Supplementary to Fig. 4. This file was assembled at 6 frames/second. Click here for file

Additional File 5 Rotating 3D projection of the branchial region of a live CAG::H2B-EGFP Tg/+ E10.5 embryo. Supplementary to stills presented in Fig. 4. This file was assembled at 6 frames/second. Click here for file

Additional File 6 Time-lapse sequence of CAG::H2B-EGFP Tg/+ ES cells. Supplementary to stills presented in Fig. 6. This file was assembled at 12 frames/second. Click here for file

Additional File 7 Time-lapse sequence of CAG::H2B-EGFP Tg/+ preimplantation embryos. Supplementary to stills presented in Fig. 6. This file was assembled at 6 frames/second. Click here for file

Additional File 8 Time-lapse sequence of a CAG::H2B-EGFP Tg/+ postimplantation embryo. Supplementary to stills presented in Fig. 6. This file was assembled at 6 frames/second. Click here for file

Additional File 9 Rotating 3D projection of a fixed CAG::H2B-EGFP Tg/+ E8.5 embryo. Supplementary to stills presented in Fig. 7. This file was assembled at 6 frames/second. Click here for file

Additional File 10 High-magnification rotating 3D projection of the headfold region of the same fixed CAG::H2B-EGFP Tg/+ E8.5 embryo as shown in Movie 7. Supplementary to stills presented in Fig. 7. This file was assembled at 6 frames/second. Click here for file

Additional File 11 High-magnification rotating 3D projection of the allantois and posterior tail region of the same fixed CAG::H2B-EGFP Tg/+ E8.5 embryo as shown in Movie 7. Supplementary to stills presented in Fig. 7. This file was assembled at 6 frames/second. Click here for file
